# Draft genome sequence of *Staphylococcus aureus* M32 isolated from a cow with subclinical mastitis in Kiruhura District, Uganda

**DOI:** 10.1128/mra.00096-26

**Published:** 2026-06-11

**Authors:** Reagan Muhwezi, Vidya Sankarapandian, G. R. Neel, Emmanuel Eilu, Ambrose Shabohurira, Ibrahim Ntulume, Lutaaya Festus, Priscilla Peter Dilli, Theophilus Pius, Naheem Adekilekun Tijani, Danladi Makeri

**Affiliations:** 1 Department of Microbiology and Immunology, Kampala International University Western Campus 365672, Bushenyi, Western Uganda; 2 Institute of Allied Health Sciences, Clarke International University 127813, Kampala, Uganda; 3 College of Veterinary Medicine, Animal Resources and Biosecurity (CoVAB), Makerere University 58588, Kampala, Central Region, Uganda; 4 Department of Public Health, Kampala International University, Western Campus 365672, Ishaka, Uganda; 5 Department of Medical Laboratory Science, Kampala International University Western Campus 365672, Ishaka, Uganda; University of Maryland Baltimore School of Medicine 12264, Baltimore, Maryland, USA

**Keywords:** complete genome, antimicrobial-resistant genes, subclinical mastitis, genomic annotation, *Staphylococcus aureus* strain 32M, Kiruhura district, Uganda

## Abstract

We report the draft genome sequence of *Staphylococcus aureus* strain M32, isolated from a cow with subclinical mastitis in Kiruhura district, Uganda.

## ANNOUNCEMENT

Subclinical mastitis (SCM) poses a threat to the dairy industry in low-income nations, resulting in food insecurity and financial losses to dairy farmers (1). Studies have linked *Staphylococcus aureus* with the disease through the milking process (2). This genome data set provides genomic insights into putative resistance and virulence genes of *S. aureus* strain M32, offering valuable information for researchers studying antibiotic stewardship, disease evolution, and genetic surveillance.

A milk sample obtained from a cow in Kiruhura District, Uganda (0°12′53.0″S, 30°46′12.0″E), was tested using the California Mastitis Test for SCM, and the positive sample was cultured on blood agar. The isolate was further sub-cultured on mannitol salt agar (MSA) (HiMedia, India) and incubated aerobically for 24 h at 37°C. Yellow colonies suggestive of *S. aureus* were purified on fresh MSA plates and verified using positive catalase, coagulase, and Staphaurex latex agglutination assays (3). Antibiotic susceptibility testing was conducted using the Kirby-Bauer, standard disk diffusion method guided by the CLSI guidelines (4, 5). The isolate was resistant to penicillin (10 μg; 6 mm), erythromycin (15 μg; 10 mm), tetracycline (30 μg; 12 mm), and cefoxitin (30 μg; 18 mm).

Strain M32 isolate was cultured in Luria-Bertani broth, incubated in a shaker at 200 rpm for 18–24 h at 37°C, and DNA was extracted using a column-based extraction with the NEB Monarch Spin gDNA Extraction Kit, including the extra lysozyme pre-treatment step for efficient lysis of gram-positive cells (6). DNA concentration and purity were measured using the Qubit 3.0 Fluorometer (Thermo Fisher Scientific) with Quant-iT dsDNA Assay Kit, High Sensitivity (Invitrogen), and NanoDrop ND 1000 spectrophotometer. Rapid barcoding kit 96V14 (SQK-RBK114.96) was used for library preparation and loaded onto an R10 flow cell (FLO-MIN114) (7). The MinION MK1B (MIN-101B) platform was used for draft genome sequencing (8). Guppy (v6.5.7-gpu) with a super-accurate base caller was used to translate the raw ionic current signals into nucleotide sequences (1D reads) (9).

Raw sequence reads were assessed for quality using NanoPlot (v1.46.0) (10), before being trimmed, yielding 16,784.0 reads, a read length N50 of 207.0 bp, and 3,619,882.0 bp bases, and after trimming, yielding 1,104.0 reads, a read length N50 of 1546.0 bp, and 1,458,609.0 bp bases. Adapter sequences and low-quality reads were trimmed using NanoFilt (v2.8.0) (10), and only high-quality reads were retained for downstream analysis. *De novo* genome assembly was performed using Flye (v2.9.5-b180) (11), followed by consensus polishing with Medaka. Assembly quality was evaluated using QUAST (v5.3.0) (12). Genome completeness was assessed using BUSCO (v5.8.3) (13), yielding 91.3% complete BUSCOs (90.7% single-copy, 0.5% duplicated), with 2.5% fragmented and 6.2% missing genes (*n* = 1,317). The assembled genome was submitted and annotated using the National Center for Biotechnology Information (NCBI) Prokaryotic Genome Annotation Pipeline (v6.10) (14). The assembled genome size yielded was 2,765,223 bp with a 33% GC, 294 contigs, 14.8 kb N50, and 50 L50. For the submitted genome, CheckM (v1.2.4) quality analyzer revealed 89.67% completeness and 1.93% contamination (15). Average Nucleotide Identity (ANI) analysis using FastANI (v1.34) revealed that the closest type strain to our isolate was *Staphylococcus aureus* subsp. *aureus* DSM 20,231 (GCA_000330825.2) with an ANI of 99.04%, assembly coverage of 89.46%, and type assembly coverage of 89.75%, conﬁrming species-level identity and close relatedness in NCBI (16). Other genome characteristics are presented in Table 1 below.

**TABLE 1:** Genome statistics and features of *S. aureus* strain M32

Parameter	Value
Genes	2,927
CDSs	2,873
Ribosomal RNAs	2, 1, 1 (5S, 16S, 23S)
Transfer RNAs	46
Non-coding RNAs	4

## Data Availability

The draft genome has been submitted to NCBI GenBank under accession number JBQVMH000000000.1 (17), with BioSample number SAMN50774900 (18). Raw sequence reads and annotated genome are available under accessions SRR35120694 (19) and GCA_052439135.1 (20), respectively.
